# Cholera outbreak caused by drinking lake water contaminated with human faeces in Kaiso Village, Hoima District, Western Uganda, October 2015

**DOI:** 10.1186/s40249-017-0359-2

**Published:** 2017-10-10

**Authors:** David W. Oguttu, A. Okullo, G. Bwire, P. Nsubuga, A.R. Ario

**Affiliations:** 1 Uganda Public Health Fellowship Program – Field Epidemiology Track, P.O. Box 7272, Kampala, Uganda; 2 Ministry of Health, Kampala, Uganda; 3 African Field Epidemiology Network, Kampala, Uganda

**Keywords:** Cholera, Outbreak, Faeces, Lake water, Uganda

## Abstract

**Background:**

On 12 October 2015, a cholera outbreak involving 65 cases and two deaths was reported in a fishing village in Hoima District, Western Uganda. Despite initial response by the local health department, the outbreak persisted. We conducted an investigation to identify the source and mode of transmission, and recommend evidence-led interventions to control and prevent cholera outbreaks in this area.

**Methods:**

We defined a suspected case as the onset of acute watery diarrhoea from 1 October to 2 November 2015 in a resident of Kaiso Village. A confirmed case was a suspected case who had *Vibrio cholerae* isolated from stool. We found cases by record review and active community case finding. We performed descriptive epidemiologic analysis for hypothesis generation. In an unmatched case-control study, we compared exposure histories of 61 cases and 126 controls randomly selected among asymptomatic village residents. We also conducted an environmental assessment and obtained meteorological data from a weather station.

**Results:**

We identified 122 suspected cases, of which six were culture-confirmed, 47 were confirmed positive with a rapid diagnostic test and two died. The two deceased cases had onset of the disease on 2 October and 10 October, respectively. Heavy rainfall occurred on 7–11 October; a point-source outbreak occurred on 12–15 October, followed by continuous community transmission for two weeks. Village residents usually collected drinking water from three lakeshore points – A, B and C: 9.8% (6/61) of case-persons and 31% (39/126) of control-persons were found to usually use point A, 21% (13/61) of case-persons and 37% (46/126) of control-persons were found to usually use point B (*OR* = 1.8, 95% *CI*: 0.64–5.3), and 69% (42/61) of case-persons and 33% (41/126) of control-persons were found to usually use point C (*OR* = 6.7; 95% *CI*: 2.5–17) for water collection. All case-persons (61/61) and 93% (117/126) of control-persons reportedly never treated/boiled drinking water (*OR* = ∞, 95% *CI*_Fisher_: 1.0 – ∞). The village’s piped water system had been vandalised and open defecation was common due to a lack of latrines. The lake water was found to be contiminated due to a gully channel that washed the faeces into the lake at point C.

**Conclusions:**

This outbreak was likely caused by drinking lake water contaminated by faeces from a gully channel. We recommend treatment of drinking water, fixing the vandalised piped-water system and constructing latrines.

**Electronic supplementary material:**

The online version of this article (10.1186/s40249-017-0359-2) contains supplementary material, which is available to authorized users.

## Multilingual abstracts

Please see Additional file 1 for translations of the abstract into the five official working languages of the United Nations.

## Background

Cholera is a diarrhoeal disease of epidemic potential caused by gram-negative bacteria, *Vibrio cholerae* [1, 2]. The pathogen produces a toxin that causes severe loss of water and electrolytes in patients through diarrhoea [3]. The disease has an incubation period of a few hours to 5 days [2]. The main modes of transmission are consumption of the bacteria in food or water [4, 5].

The global burden of cholera is often underestimated due to a lack of systematic epidemiologic studies being conducted in endemic countries, inadequate outbreak investigation and laxity of reporting cholera outbreaks by some countries out of fear of economic consequences [1, 4, 6].

It is estimated that out of 1.3 billion people at risk of cholera globally, 2.86 million cholera cases occur annually in endemic countries resulting in 95,000 deaths, with the majority of these in Africa [5]. Cholera has been described as one of the diseases associated with poverty [7]. Urban slums and fishing communities in African countries are at greater risk of cholera epidemics because of overcrowding, poor sanitation and lack of a safe water supply [8–13]. Implementation of key public health strategies to achieve cholera prevention and eventual elimination is still lacking in many developing countries in Africa and Asia [14].

Since 1997, cholera outbreaks have occurred in Uganda annually, with an average of 11,000 cases and 60–182 deaths per year [15, 16]. Rural areas neighbouring the Democratic Republic of the Congo are prone to cholera outbreaks due to the interaction of people from different traditional settings with diverse hygiene and sanitation practices [15]. Lakeside and slum areas of Uganda are are known to be hotspots of cholera outbreaks during heavy rainfall [13, 17]. Poor environmental sanitation, lack of safe water and trans-regional travel are among the key factors contributing to the spread and sustainment of cholera outbreaks in the country [18]. In some outbreaks, causative agents are isolated, but field investigations that generate and test hypotheses to identify the source of infection to guide specific control measures are generally lacking [13]. General public health interventions such as health education, closing restaurants and market vendors abolishing beverage sales have been used as interventions to control cholera outbreaks, causing undue inconvenience to the business community and often failing to stop and prevent future outbreaks in endemic areas [19]. In September 2016, the Ministry of Health responded to a cholera outbreak in Nkondo, a village located 5 km from Kaiso.

On 12 October 2015, the Health Officer of Hoima District, Western Uganda reported an outbreak of cholera in Kaiso Village on the shoreline of Lake Albert, following isolation of *V. cholerae* from stool samples of three suspected cases by the Central Public Health Laboratory (CPHL). Two people died and many others were ill with similar symptoms. The district and the Ministry of Health responded by setting up a cholera treatment centre (CTC) in the village and conducting health education in the community. However, the response did not stop the outbreak, and there was a need for a detailed epidemiological investigation to recommend specific interventions. The Ministry of Health requested the fellows of the Uganda Public Health Fellowship Program – Field Epidemiology Track to conduct an epidemiological investigation. The purpose of the investigation was to identify the source and mode of transmission of the cholera outbreak, and recommend evidence-based interventions to stop the outbreak and prevent future outbreaks. This paper describes this investigation.

## Methods

### Study area

The study area, Kaiso Village, is located in Buseruka Sub-County on the shoreline of Lake Albert in Hoima District, Western Uganda. The village consists of three settlement zones (SZs): Fichama (SZ1), Songa-Bakobya (SZ2) and Songa-Lendu (SZ3).

Kaiso has a sloping landscape extending from the escarpment of the western rift valley down to the shoreline of Lake Albert. SZ3 has mostly hillside homesteads. The village has three water collection sites on the shoreline of Lake Albert at the edge of each zone.

**Fig. 1:**
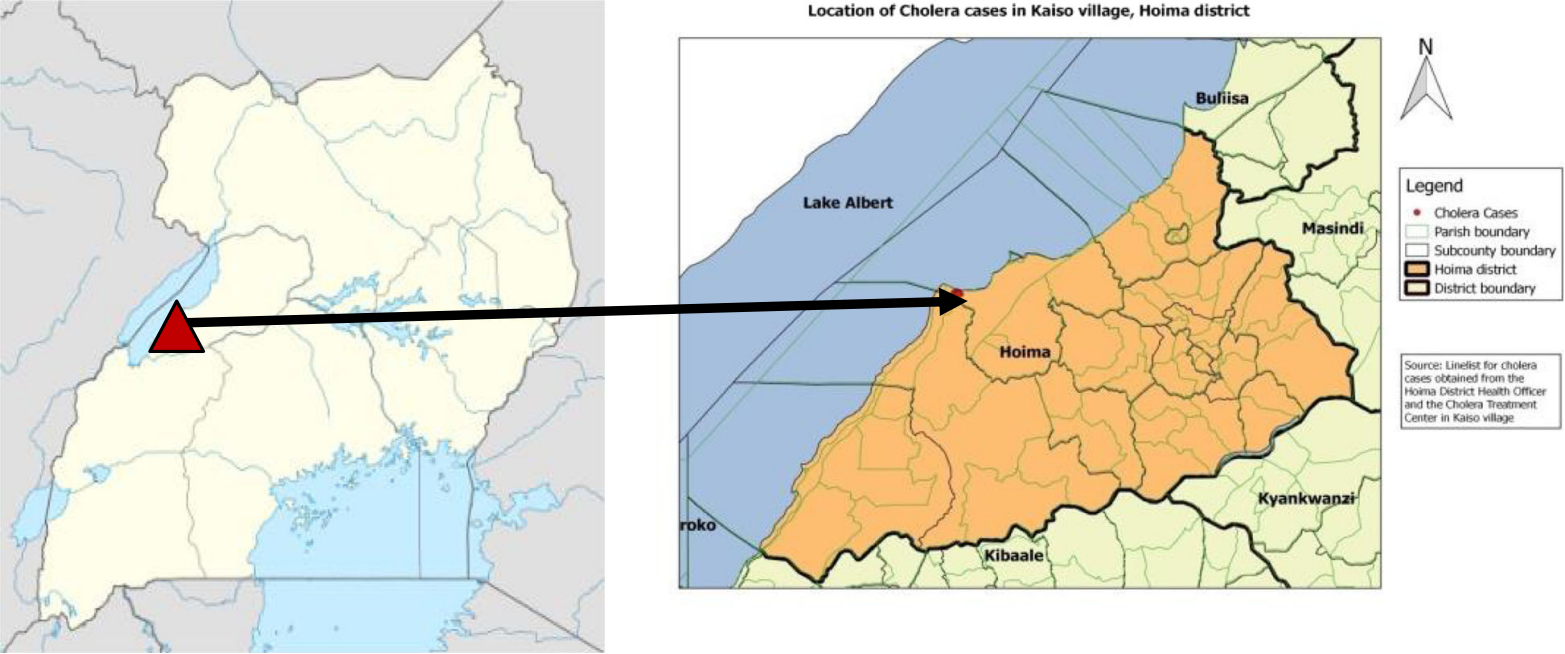
Location of Kaiso Village, Hoima District, Western Uganda. The village consists of three settlement zones (SZ): Fichama (SZ1), Songa-Bakobya (SZ2) and Songa-Lendu (SZ3)

The entire village had a population of approximately 9000 people according to the village register of September 2015 (500 in SZ1, 2000 in SZ2 and 6500 in SZ3). The major economic activities are fishing and small-scale trading. A large proportion of the community is comprised of mobile people who camp in shoreline villages looking for better fish catches, as well as traders who buy and transport fish and other merchandise to markets in Uganda and Congo (see Fig. 1).

### Case definition

We defined a suspected case as the onset of acute watery diarrhoea from 1 October to 2 November 2015 in a resident of Kaiso Village. A confirmed case was a suspected case with *V. cholerae* isolated from a stool specimen.

### Finding and interviewing cases

We visited the CTC that was set up in the village and used records to list cases by the date of admission. With the guidance of village health teams, we found and interviewed more cases by visiting households in the village. Using a case investigation questionnaire, we obtained demographic and epidemiological information on symptoms, date of onset and key exposures for descriptive analysis. Out of the 122 case-persons line listed, we interviewed the initial 57 about water and food consumption within 5 days of onset of symptoms.

### Verification of diagnosis

We transported stool samples from 10 suspected cases in Cary-Blair medium to the CPHL for isolation of *V. cholerae* by culture. Stool samples of subsequent cases were screened using the rapid immunochromatographic diagnostic test Crystal VC™ dipstick (Span Diagnostics Ltd., Surat, India) at the CTC. Results were read after incubation for 15 min following the manufacturer’s instructions, using the standard procedure described by Nato et al. [20, 21].

### Descriptive epidemiology and hypothesis generation

We analysed the data gathered from case investigation by time, place and person to generate a hypothesis for our investigation. Findings of our descriptive epidemiology suggested that all cases drunk untreated lake water before the onset of illness. We hypothesised that drinking lake water was the likely cause of the outbreak.

### Case-control study

We conducted an unmatched case-control study to test the hypothesis, in which we interviewed 61 suspected cases and 126 controls using a structured questionnaire. We estimated the sample size by assuming a confidence level of 95%, 80% power, 50% exposure of controls to drinking lake water and an odds ratio (*OR*) of at least 2.5, as described in a study on similar lakeshore outbreak [22]. We only selected one case per household if there were multiple cases in the same household. We excluded suspected cases who were < 5 years old.

Controls were residents of Kaiso Village aged ≥ 5 years without the onset of acute watery diarrhoea from 1 October 2015 to 3 November 2015. Controls were selected from the village register using simple random sampling.

Three village health workers were trained to administer questionnaires to cases and controls in their local languages. We asked case- and control-persons about their sources of drinking water, food and history of boiling or treating drinking water a week before the outbreak.

### Environmental assessment

On suspicion that drinking water might have been the cause of this outbreak based on the descriptive epidemiology analysis, we inspected three shoreline water collection sites – A, B and C – in the village to investigate how the drinking water might have become contaminated. We used Google Maps to draw a map of the village area to show the major source of contamination and distribution of cholera case households. We also observed practices of human faecal disposal in the village, and assessed availability of safe water sources and latrine coverage. We obtained rainfall data around the outbreak period from a local weather station located 2 km from the village.

### Data analysis

We performed univariate analysis to assess the associations between exposure and the disease. We also conducted a stratified analysis for exposures with significant ORs on univariate analysis to obtain Mantel-Haenszel adjusted *OR*s (*OR*_M-H_) and their corresponding 95% confidence intervals (*CI*s) [23].

### Ethical considerations

The Ministry of Health of Uganda gave the directive and approval to investigate this outbreak. The Office of the Associate Director for Science at the Center for Disease Control and Prevention (CDC) Uganda determined that this research did not involve human subjects and that its primary intent was public health practice or disease control activity (specifically, epidemic or endemic disease control activity). Verbal informed consent was obtained from the participants or, if the interviewee was a minor, guardians before the start of each interview.

## Results

### Descriptive epidemiology and hypothesis generated

We identified 122 cases, including two deaths that were registered on 2 October and 10 October, respectively. The index case-person was an adult fisherman who died in the community after suffering from diarrhoea and vomiting. The primary case-person in this outbreak was not identified.

The highest attack rate was in SZ2 and the least was in SZ1. However, most of the cases occurred in SZ3 (see Table 1).

**Table 1:** Cholera attack rate by SZ during an outbreak in Kaiso Village, Hoima District, Western Uganda, October – November 2015

		Population	Attack rate
SZ	Cases	*(estimated)*	*(per 1000)*
2 (Songa-Bakobya)	35	2000	18
3 (Songa-Lendu)	85	6500	13
1 (Fichama)	2	500	4.0

Of the 122 cases, 70 (57%) were males and 52 (43%) were females. Both children and adults were affected (median age of case-persons = 20 years; range: 1–65 years). Of the 122 cases, 39 (32%) were fishermen and the rest had other occupations.

Of the cases identified, 98% depended on lake water and 100% drunk it unboiled. Most of the case-persons ate hot food in their homes.

Based on the results of the descriptive epidemiology, we hypothesised that drinking contaminated water from one of the lakeshore water collection sites caused this outbreak.

### Findings of the case-control study

Residents who collected their drinking water from site C (in SZ3 area) were significantly more likely to develop cholera compared to those who collected their drinking water from site A (in SZ1) (*OR*_M-H_ = 6.7, 95% *CI*: 2.5–17); collecting drinking water from site B (in SZ2) was not significantly associated with an increased risk of developing choleara. In addition, people who drank unboiled water were most likely to get cholera (*OR*_M-H_ = ∞, 95% *CI*_Fisher’s exact_: 1.0 – ∞) (see Table 2).

**Table 2:** Association between water exposure and development of cholera during an outbreak in Kaiso Village, Hoima District, Western Uganda, October – November 2015

Exposure	% exposed		*OR*_M-H (95% *CI*)_
	Cases*(n = 61)*	Controls*(n = 126)*	
Usual water collection site			
C (Songa-Lendu)	69	33	6.7 (2.5–17)
B (Songa-Bakobya)	21	37	1.8 (0.64–5.3)
A (Lake-Rescue A)	10	30	Ref.
Boiling water before drinking			
No	100	97	∞ (1.0 – ∞)^a^
Yes	0	7	Ref.

^a^ *Fisher’s exact CI*

### Environmental assessment

**Fig. 2:**
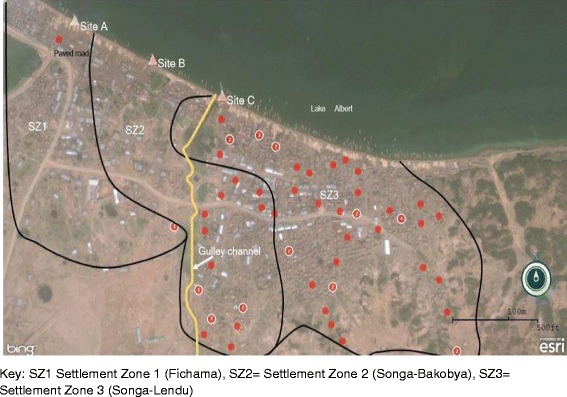
Case-cluster map showing distribution of households of cholera case-patients during an outbreak in Kaiso Village, by SZ, water collection site and gulley channel. A gully channel opens into the lake near water collection site C. Open defecation was common along the gully channel due to a lack of pit latrines in 90% of the households and the entire village had one public latrine, which was not functional. Human faeces from the open defecation area are washed into the lake near site C through the gully channel when precipitation occurrs

Three water collection sites (A, B and C) were identified along the shoreline. Site A, known as the ‘Lake Rescue’, was protected from run-off contaminants by a paved road constructed parallel to the shoreline. Residents of SZ1 usually collected water from site A. Site B was partially protected by a paved portion of the road. Site C was where residents of SZ2 and SZ3 collected their water from (see Fig. 2).

**Fig. 3:**
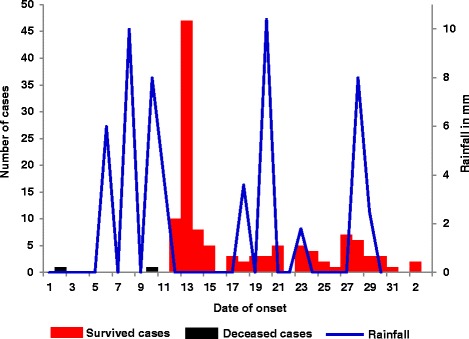
Onset dates of cholera case-patients in relation to rainfall during a cholera outbreak in Kaiso Village, Hoima District, Western Uganda, October – November 2015. The epidemic curve shows that after the onset of disease on 2 October and 10 October, respectively, in the two initial deceased cases, there was heavy rainfall on 7–11 October. An ensuring point-source outbreak occurred on 12–15 October, followed by continuous community transmission for two weeks. The outbreak ended on 3 November 2015

Meteorological data showed that the peak of the cholera outbreak occurred in the community following heavy rainfall. After a community awareness campaign and distribution of chlorine to households, the number of cases reduced despite continued rainfall (see Fig. 3).

The village used to have a piped water system powered by solar panels. This system used to be the only source of safe water in the village. However, the system was vandalised 6 months prior to the outbreak and was never repaired, forcing the community to depend on the lake as the primary source of drinking water.

### Laboratory results

*V. cholerae* was isolated from six of the 10 stool samples submitted to the CPHL to confirm the outbreak. Of the 59 patients tested with the cholera rapid diagnostic test in the CTC, 47 were found to be positive for *V. cholerae* 01.

## Discussion

Our investigation demonstrated that the cholera outbreak in Kaiso Village was caused by drinking contaminated lake water. Prior to this outbreak, cholera outbreaks had been reported in communities along the Lake Albert shoreline. Hence, cholera might have been introduced into the Kaiso community by one or multiple visitors carrying the bacteria, causing the initial infections. Those initial case-patients likely had defecated on the hillside area along the gulley channel. Heavy rainfall subsequently washed the case-patients’ faeces down the gulley channel onto the lakeshore. Village residents collected the contaminated lake water near the end of the gully channel and drank it without boiling or treating it, causing the outbreak.

Since 1997, cholera outbreaks have occurred in different regions of Uganda every year [15]. The *V. cholerae Ogawa* serotype has been isolated in over 80% of the outbreaks. Fishing communities around Lake Albert and town slums are among the major outbreak-prone areas in the country. Most of these outbreaks have not been investigated epidemiologically, and have subsided and eventually stopped after the implementation of general interventions. Inadequate investigations on how these outbreaks occurred and hence the lack of specific-evidence based interventions might have been one of the reasons why cholera outbreaks, such as the current outbreak, have repeatedly occurred in fishing villages and slum residential areas.

Persistent outbreaks of water-borne diseases such as cholera in Africa have been attributed to overcrowding, poor sanitation and lack of a safe water supply [8–13]. Commitment to implementating key public health interventions to prevent and eliminate water-borne diseases is still a challenge in many countries in Africa and Asia [14]. Our investigation has reaffirmed findings from previous investigations that a lack of safe water [9–11, 19] and poor sanitation [13, 17, 18] are the major causes of cholera outbreaks in resource-limited settings.

Unlike in previous cholera outbreaks in Uganda, which did not ascertain the mode of transmission, our investigation pinpointed the contamination point, identified how the lakeshore water had likely become contaminated and how case-patients had been exposed to the contaminated water. The information collected from the investigation informed the district health officials to implement immediate and long-term interventions to stop the current outbreak and to prevent future outbreaks of cholera and other water-borne diseases.

The case-finding in the initial phase of this outbreak investigation relied on the data collected at the CTC, which was established after 12 October. The data showed that prior to October 11, only two case-patients were reported; both died. Historically, the case-fatality ratio for cholera has ranged from < 1% with adequate management to as high as 50% when untreated [2, 24, 25]. Therefore, one would expect that with two deaths, there might have been anywhere from several to scores of cases that were never registered at the CTC, which might explain the sharp increase in the number of cases on 13 October.

## Conclusions

Our investigation found that the cholera outbreak in Kaiso Village was caused by drinking untreated lake water contaminated by human faeces washed down a gully channel during heavy rainfalls. We recommend treatment and boiling of drinking water to stop the outbreak. We also recommend fixing the vandalised piped water system and protecting the solar panels and equipment by placing wires and guards around the facility, and constructing latrines in the community, to prevent future cholera outbreaks.

This study demonstrates the importance of conducting a complete outbreak investigation in a developing country to get epidemiological evidence to guide specific public health actions to stop an outbreak, as well as to generate recommendations to prevent similar events occuring in future.

## Additional file

Additional file 1: Multilingual abstracts in the five official working languages of the United Nations. (PDF 659 kb)

## Abbreviations

CDC: Center for Disease Control and Prevention
*CI*: Confidence interval
CPHL: Central Public Health Laboratory
CTC: Cholera treatment center
*OR*: Odds ratio
*OR*_M-H_: Mantel-Haenszel adjusted *OR*s
SZ: Settlement zone
