# Supplementary material for: Cholera outbreak caused by drinking lake water contaminated with human faeces in Kaiso Village, Hoima District, Western Uganda, October 2015
Source: Infect Dis Poverty. 2017 Oct 10;6:146. doi: 10.1186/s40249-017-0359-2 (PMC5634859; doi:10.1186/s40249-017-0359-2)
Supplement: Additional file 1: — Multilingual abstracts in the five official working languages of the United Nations. (PDF 659 kb) [file 40249_2017_359_MOESM1_ESM.pdf]

Translation of the abstract into the five official working languages of the United Nations

تفشي وباء الكوليرا الناجم عن شرب المياة الملوثة بالبراز البشري من بحيرة في قرية كايسو في منطقة هويما بغرب أوغندا، تشرين الأول/أكتوبر عام 2015

David W. Oguttu, A. Okullo, G. Bwire, P. Nsubuga and A.R. Ario

ملخص

خلفية: وفي 12 تشرين الأول / أكتوبر 2015، جاء بلاغ بتفشي وباء الكوليرا ليصيب 65 شخص ووفاة اثنان آخرون ارصدت في قرية صيد، مقاطعة هويما،غرب أوغندا. واستمر تفشي المرض على الرغم من الاستجابة الأولية لإدارة الصحة المحلية. أجرينا تحقيقا ل تحديد مصدر وطريقة انتقال العدوى،والتوصيات الموصي بها للوصول للأدلة للسيطرة على تفشي وباء الكوليرا ومنع حدوثه في المنطقة.

الطرق: حددنا حالة من الإسهال المائي الحاد مشتبهٌ فيها من 1 تشرين الأول / أكتوبر إلى 2 تشرين الثاني / نوفمبر 2015 في أحد سكان قرية كايسو. ا إن الحالة المؤكدة اصبحت مشتبه بها حيث *تم عزل الهيضة الكوليرية من البراز* وبمراجعة السجلات والبحث النشط في المجتمع وجدنا الحالات. أجرينا تحليل وصفي وبائي لتوليد الفرضيات. في دراسة غير مسبوقة للسيطرة على الحالات، قمنا بمقارنة تاريخ تعرض 61 حالة و 126 من الضوابط التي تم اختيارها عشوائيا من بين سكان القرية (بدون أعراض). كما أجرينا تقييما بيئيا وحصلنا على بيانات الأرصاد الجوية من محطة الطقس.

النتائج: حددنا 122 حالة يشتبه في إصابتهامن خلال اختبار تشخيصي سريع، منها ستة حالات مؤكدة مبدئيًا، و 47 حالة مؤكدة تمامًا وتوفي اثنان منهم. وتوفيت حالتين بعد الأصابة بالمرض في 2 تشرين الأول / أكتوبر و 10 تشرين الأول / أكتوبر على التوالي. ووقعت أمطار غزيرة في 7 - 11 تشرين الأول / أكتوبر؛ حدث تفشي في 12-15 أكتوبر، تليها انتقال مجتمعي مستمر لمدة أسبوعين. وقد وجد أن سكان القرية عادة ما يستخدمون مياه الشرب من ثلاث نقاط على شاطئ البحيرة - A و B و C: 9.8٪ (61/6) من الأشخاص و 31٪ (126/39) من الأشخاص الخاضعين للمراقبة كانوا يستخدمون عادة النقطة A، و 21٪ (61/13) من الأشخاص المعينين و 37٪ (126/46) من الأشخاص الخاضعين للمراقبة يستخدمون عادة النقطة B (أو = 1.8، 95٪ *CI*: 0.64 - 5.3)، و 69٪ (61/42) من الحالات و 33٪ (126/41) من الأشخاص الخاضعين للمراقبة يستخدمون عادة النقطة C (أو = 6.7؛ 95٪ *CI*: 2.5 - 17) لجمع المياه. وأفيد بأن جميع الأشخاص (61/61) و 93 في المائة (126/117) من الأشخاص الخاضعين للمراقبة لم يعالجوا او يغلو مياه الشرب قط (أو = ∞، 95٪ $CI_{\text{الصياد}}$: 1.0 - ∞). وقد تم تخريب نظام انابيب المياه في القرية، وكان التغوط المفتوح شائعا بسبب عدم وجود مراحيض. حيث من المفترض أن مياه البحيرة يتم تنقيتها من خلال قناة التوليد التي نظفت البراز في البحيرة في النقطة C.

الاستنتاجات: ومن المحتمل أن يكون هذا ناجمًا عن شرب مياه البحيرة الملوثة بالبراز من مجرى القناة. ونوصي بمعالجة مياه الشرب، وتثبيت نظام الانابيب وتنقية المياه، وإنشاء المراحيض.

Translated from English version into Arabic through

TRANSLATORS
WITHOUT BORDERS

2015 年 10 月在乌干达西部的 Kaiso 地区 Kaiso 村因饮用被人类粪便污染的湖水而引发的霍乱疫情

David W. Oguttu, A. Okullo, G. Bwire, P. Nsubuga and A.R. Ario[1]

摘要

引言：2015 年 10 月 12 日，在乌干达西部 Hoima 地区的一个渔村报告了 65 例霍乱病例和 2 例死亡病例。尽管当地卫生部门积极应对，但疫情仍在持续。我们对该次霍乱疫情进行调查以确定传播来源和模式，并建议采取以证据为导向的干预措施，控制和预防该地区的霍乱疫情。

方法：我们将 2015 年 10 月 1 日至 11 月 2 日，在 Kaiso 村发生急性水样腹泻的病例定义为

疑似病例。从粪便中分离出霍乱弧菌（*Vibrio cholerae*）的疑似病例确定为确诊病例。我们通过记录审查和主动的社区搜索来发现病例。对假定发生的病例进行了描述性流行病学分析。在一项不匹配的病例对照研究中，我们比较了 61 例病例，以及从无症状村居民中随机选择的 126 例对照的暴露史。此外，还进行了环境评估，并从气象站获得气象数据。

结果：共确认 123 例疑似病例，其中 6 例经细菌培养确诊，47 例经快速检测试验确诊为阳性，2 例死亡。两例死亡病例分别于 10 月 2 日和 10 月 10 日发病。10 月 7-11 日当地下大雨，10 月 12-15 日发生点源爆发，随后在社区传播持续了两周。村民通常从 A、B 和 C 三个湖岸点收集饮用水。通常于 A 点取水的病例和对照为 9.8%（6/61）和 31%（39/126），B 点为 21%（13/61）和 37%（46/126）（*OR* = 1.8, 95%*CI*：0.64-5.3），C 点为 69%（42/61）的病例和 33%（41/126）（*OR* = 6.7; 95%*CI*：2.5-17）。所有病例（61/61）和 93%（117/126）的对照从未对饮用水做过处理（*OR* =∞，95%CI_{Fisher}：1.0-∞）。因厕所数量不足，开放排便很普遍，同时该村的自来水供水系统破损。C 点粪便经沟槽流入湖中造成湖水污染。

结论：该次霍乱暴发疫情很可能是因人们饮用了被粪便污染的湖水而引起的。因此，我们建议对饮用水进行处理，修复破损的自来水系统，并修建厕所。

Translated from English version into Chinese by Xin-Yu Feng, edited by Pin Yang

CHINESE CENTER FOR DISEASE CONTROL AND PREVENTION
NATIONAL INSTITUTE OF PARASITIC DISEASES

Éclosion de choléra, causée par la consommation d'eau de lac contaminé par des excréments humains dans le village de Kaiso, dans le district de Hoima, en Ouganda occidental, octobre 2015.

David W. Oguttu, A. Okullo, G. Bwire, P. Nsubuga et A.R. Ario

Résumé

Le contexte : Le 12 octobre 2015, une éclosion de choléra, impliquant 65 cas et deux décès, fut rapportée dans un village côtier du district d'Hoima, en Ouganda occidental. Malgré l'intervention initiale des autorités de santé locales, l'éclosion persista. Nous avons mené une enquête pour identifier la source et le mode de transmission, puis nous recommandons certaines interventions, suivant notre collecte d'évidence, pour contrôler et prévenir l'éclosion de choléra dans ce secteur.

Les méthodes : Nous avons défini un cas suspect comme étant le début d'une diarrhée aqueuse aiguë survenue entre le 1er octobre et le 2 novembre 2015, chez un résident du village Kaiso. Uncas suspecté s'est confirmé comme étant un cas de*Vibrio cholerae*, bactérie isolée dans les selles. Nous avons trouvé des cas en examinant des dossiers et en étudiant activement certains cas dans la communauté. Nous avons effectué une analyse épidémiologique descriptive pour formuler des hypothèses générationnelles. Dans une étude de cas témoins inégalée, nous avons comparé les antécédents d'exposition de 61 cas et de 126 témoins choisis au hasard parmi les villageois asymptomatiques. Nous avons aussi procédé à une évaluation environnementale et avons obtenu des données météorologiques d'une station météo.

Les résultats : </bx>nous avons identifié 122 cas suspects, dont six ont été confirmés par cultures bactériennes, 47 ont été confirmés positivement à la suite d'un test de diagnostic rapide et deux sont décédés. Les deux cas de décès sont liés au commencement de la maladie, le 2 octobre et le 10 octobre, respectivement. Des pluies abondantes sont tombées du 7 au 11 octobre ; une éclosion de source ponctuelle s'est produite entre le 12 et le 15 octobre, suivie par une transmission collective

continuelle pendant deux semaines. Les villageois recueillaient habituellement l'eau potable à trois points précis sur la rive du lac, c'est-à-dire aux points A, B et C. Il a été démontré que 9,8 % (6/61) des victimes et 31 % (39/126) des cas témoins recueillaient leur eau au point A. Alors que 21 % (13/61) des victimes et 37 % (46/126) des cas témoins prenaient habituellement leur eau au point B (*ou*= 1,8, 95 %*CI* : 0,64 – 5,3). Tandis que 69 % (42/61) des victimes et 33 % (41/126) des cas témoins prenaient habituellement leur eau au point C (*ou* = 6,7 ; 95 % *CI* : 2,5 – 17) . Toutes les victimes (61/61) et 93 % (117/126) des cas témoins n'auraient jamais traité ni fait bouillir leur eau potable (*ou* = ∞, 95 % CI_{Fisher}: 1,0 – ∞). Le système d'eau courante du village avait été vandalisé et il était fréquent de retrouver de la déjection due à un manque de latrines. Nous avons constaté que l'eau du lac était contaminée par les selles provenant d'un puisard rejeté au point C du lac.
En conclusions :</bx> cette éclosion a sans doute été provoquée par l'eau potable du lac contaminée par les selles provenant d'un puisard. Nous recommandons le traitement de l'eau potable, la réparation du système d'aqueduc vandalisé et la construction de latrines.

Translated from English version into French by Nathalie Thompson, through

TRANSLATORS
WITHOUT BORDERS

Вспышка холеры, вызванная питьем озерной воды, загрязненной человеческими фекалиями, в селе Кайсо, район Хойма, Западная Уганда, октябрь 2015 года

Давид В. Огутту, А. Окулло, Г. Бвир, П. Нсубуга и А.Р. Арио

Аннотация
Основные факты: 12 октября 2015 г. была зарегистрирована вспышка холеры с 65 заболевшими и двумя летальными исходами в рыбацкой деревне в районе Хойма в Западной Уганде. Несмотря на ответные меры, предпринятые местным департаментом здравоохранения, эпидемия продолжалась. Мы провели исследование для того, чтобы выявить источник и способ распространения болезни, а также, основываясь на полученных данных, рекомендовать меры борьбы и профилактики вспышек холеры в этом регионе.
Методы: Мы определили предполагаемый случай как заболевание острой водянистой диареей, развившееся с 1 октября по 2 ноября 2015 г. у жителя деревни Кайсо. Подозрение на холеру стало подтвержденным случаем после выделения из стула больного *Vibrio cholerae.* Мы обнаружили случаи путем анализа записей и активного эпидемиологического обследования местного населения. Мы провели описательный эпидемиологический анализ для составления гипотез. В исследовании сходных неспаренных случаев мы сравнивали истории экспозиции, включая 61 случай и 126 контрольных групп, случайно выбранных среди жителей села, у которых не было симптомов. Мы также провели оценку окружающей среды и получили метеорологические данные с метеостанции.
Результаты: Мы выявили 122 подозрительных случая, из которых шесть были подтверждены результатами посева, 47 были подтверждены при помощи положительного быстрого диагностического теста, а два окончились летальными исходами. В двух случаях, закончившихся летальными исходами, заболевание началось 2 октября и 10 октября соответственно. Сильные осадки произошли 7–11 октября; 12–15 октября произошла точечная вспышка заболевания, за которой последовала непрерывная передача инфекции внутри сообщества в течение двух недель. Обычно жители деревни брали питьевую воду из трех точек озера — A, B и C: 9,8% (6/61) пострадавших и 31% (39/126) контрольных лиц, как

правило, использовали точку А, 21% (13/61) пострадавших и 37% (46/126) контрольных лиц, как правило, использовали точку В (*ИЛИ* = 1,8, 95% *ДИ*: 0,64–5,3) и 69% (42/61) пострадавших и 33% (41/126) контрольных лиц, как правило, использовали точку С (*ИЛИ* = 6,7; 95% *ДИ*: 2,5 - 17) для сбора воды. Сообщалось, что все пострадавшие (61/61) и 93% (117/126) контрольных лиц никогда не очищали/не кипятили питьевую воду (*ИЛИ* = ∞, 95% *ДИ*$_{\text{по Фишеру}}$: 1,0 - ∞). Водопроводная система деревни была разрушена, и открытая дефекация была обычным делом из-за отсутствия туалетов. Было обнаружено, что озерная вода загрязнялась из-за ливневого канала, через который фекалии попадали в озеро в точке С.
Выводы: Эта вспышка, вероятно, была вызвана питьем озерной воды, загрязненной фекалиями из ливневого канала. Мы рекомендуем очистку питьевой воды, восстановление разрушенной водопроводной системы и строительство туалетов.

Translated from English version into Russian by Oksana Weiss, through

TRANSLATORS
WITHOUT BORDERS

Brote de cólera causado por beber agua contaminada con heces humanas en la aldea de Kaiso, Distrito de Hoima, oeste de Uganda, octubre de 2015

David W. Oguttu, A. Okullo, G. Bwire, P. Nsubuga y A.R. Ario

Resumen

Contexto: El 12 de octubre de 2015 se informó de un brote de cólera con 65 casos y dos muertes en </bx>una </bx> aldea de pescadores en el distrito de Hoima, en Uganda occidental. Pese a la respuesta inicial del departamento de sanidad local, el brote persistió. Se realizó una investigación para </ex> identificar la fuente y el modo de transmisión, así como para recomendar intervenciones basadas en pruebas con miras a controlar y prevenir brotes de cólera en esa zona.
Métodos: Se determinó un caso sospechoso como el inicio de la diarrea acuosa aguda del 1 de octubre al 2 de noviembre de 2015 en un residente de la aldea de Kaiso. Un caso confirmado se consideró sospechoso por tener *Vibrio cholerae* aisladas de las heces. Encontramos casos revisando el registro y los casos de la comunidad. Se realizó un análisis descriptivo epidemiológico para la generación de hipótesis. En un estudio de control de casos sin par se compararon el historial de exposición de 61 casos con 126 controles seleccionados al azar entre los residentes asintomáticos del pueblo. También se realizó una evaluación ambiental y se obtuvieron datos climáticos de una estación meteorológica.
Resultados: Se identificaron 122 casos sospechosos, de los cuales seis fueron confirmados por cultivo, 47 dieron positivo con una prueba diagnóstica rápida y dos murieron. Los dos fallecidos habían contraído la enfermedad el 2 de octubre y el 10 de octubre, respectivamente. Entre el 7 y el 11 de octubre se produjeron lluvias torrenciales; entre el 12 y el 15 de octubre surgió un brote de origen puntual, y en las dos semanas que siguieron, el virus continuó transmitiéndose en la comunidad. Los residentes de la aldea obtenían habitualmente el agua potable de tres puntos del lago – A, B y C: el 9,8% (6/61) de personas-caso y el 31% (39/126) de personas-control por lo general utilizaban el punto A, el 21% (13/61) de personas-caso y el 37% (46/126) de personas-control utilizaban normalmente el punto B (*O* = 1,8, 95% *CI*: 0,64 - 5,3), y el 69% (42/61) de las personas- caso y el 33% (41/126) de las personas-control utilizaban normalmente el punto C (*O* = 6,7; 95% *CI*: 2,5 - 17) para la recogida de agua. Se supo que el total de las personas de casos (61/61) y el 93% (117/126) de las personas de control nunca trataban/hervían el agua potable*O* = ∞, 95%

$CI_{Pescador}$: 1,0 - ∞). El sistema de cañerías del pueblo había sido dañado, y la práctica común era defecar al aire libre debido a la falta de letrinas. Se llegó a la conclusión de que el agua del lago estaba contaminada a causa de un sumidero que había arrastrado las heces hacia el lago en el punto C.

Conclusiones: La causa probable de este brote fue el consumo del agua del lago que estaba contaminada por las heces arrastradas por un sumidero. Se recomienda realizar un tratamiento del agua potable, reparar el sistema de tuberías dañadas y construir letrinas.

Translated from English version into Spanish by SergioLorenzi, through

TRANSLATORS
WITHOUT BORDERS
